# Global mapping of miRNA-target interactions in cattle (*Bos taurus*)

**DOI:** 10.1038/s41598-017-07880-8

**Published:** 2017-08-15

**Authors:** Troels K. H. Scheel, Michael J. Moore, Joseph M. Luna, Eiko Nishiuchi, John Fak, Robert B. Darnell, Charles M. Rice

**Affiliations:** 1 Laboratory of Virology and Infectious Disease, Center for the Study of Hepatitis C, The Rockefeller University, New York, NY USA; 2 Copenhagen Hepatitis C Program, Department of Infectious Diseases, Copenhagen University Hospital, Hvidovre, Denmark; 3 Department of Immunology and Microbiology, Faculty of Health and Medical Sciences, University of Copenhagen, Copenhagen, Denmark; 4 Laboratory of Molecular Neuro-Oncology, and Howard Hughes Medical Institute, The Rockefeller University, New York, NY USA; 5 New York Genome Center, New York, NY USA

## Abstract

With roles in development, cell proliferation and disease, micro-RNA (miRNA) biology is of great importance and a potential therapeutic target. Here we used cross-linking immunoprecipitation (CLIP) and ligation of miRNA-target chimeras on the Argonaute (AGO) protein to globally map miRNA interactions in the cow. The interactome is the deepest reported to date. miRNA targeting principles are consistent with observations in other species, but with expanded pairing rules. Experimental mapping robustly predicted functional miR-17 regulatory sites. From miRNA-specific targeting for >5000 mRNAs we determined gene ontologies (GO). This confirmed repression of genes important for embryonic development and cell cycle progress by the let-7 family, and repression of those involved in cell cycle arrest by the miR-17 family, but also suggested a number of unappreciated miRNA functions. Our results provide a significant resource for understanding of bovine and species-conserved miRNA regulation, and demonstrate the power of experimental methods for establishing comprehensive interaction maps.

## Introduction

In regulation of gene expression, miRNAs are important players in fine-tuning expression at the post-transcriptional level through mRNA translational repression and destabilization^1^. As part of the RNA-induced silencing complex (RISC), the AGO proteins engage the miRNA in a ternary complex with its target, typically mRNA 3′ UTRs. The location of miRNA target sites within the 3′ UTR, local RNA structure and competitive binding of other proteins are among the factors determining the impact of miRNA regulation^2^. Further, all binding sites in the global network compete for the same available miRNA pool. Thus, competing endogenous RNAs (ceRNA), such as other mRNAs, long non-coding RNAs, pseudogene transcripts, and circular RNAs, can impact specific miRNA binding^3, 4^.

Bioinformatic prediction of miRNA targets via algorithms, such as TargetScan, have been useful in guiding experimental efforts^5^. Predictions, however, are blind to the cellular transcriptome and the miRNA repertoire and binding patterns do not always adhere to canonical rules of base pairing. Experimental studies of miRNA interactions were enhanced by crosslinking immunoprecipitation (CLIP) methods of the AGO protein^6, 7^, where recent method improvements through direct ligation of miRNA-target chimeras permit unambiguous identification of miRNA targets^8–10^. This strategy broadened our understanding of miRNA binding landscapes, and has been used to globally map miRNA interactions in human kidney and liver cells, mouse brain and lymphocytes, and in *C. elegans* ^8–10^.

RNA centric approaches to therapy are accelerating. Treatment for hepatitis C virus by blocking a critical interaction with miR-122 has been explored in the clinic, as have miRNA perturbations for cancers and atherosclerosis^11, 12^. Therefore, detailed understanding of miRNA regulation could be important for future therapy.

For bovine disease, miRNAs also play important roles. Bovine miRNA expression in immune-related tissues and perturbation by bacterial and viral infections has been characterized^13, 14^. However, a current limitation to the translational potential of miRNA biology in cattle, however, is the lack of validated miRNAs targets^13^. Whereas miRNAs typically are tissue-specific and highly conserved between species^15^, their targetomes are not always conserved^16^. Therefore species-specific mapping is important for understanding miRNA regulation. Here, we set out to broaden the species repertoire for global miRNA landscapes by mapping interactions in bovine cells. For this, we used Madin-Darby bovine kidney (MDBK) cells, a well-established and highly used bovine cell line. Our results on miRNA binding modalities are congruent with previous reports in other species and tissues. This dataset provides a significant resource to the field, and its depth allows explorations into gene regulation not previously possible.

## Results

### CLEAR-CLIP defines the miRNA-target interaction landscape in bovine kidney cells

To globally map bovine miRNA-target interactions, we performed 39 standard AGO-CLIP^6^ or covalent ligation of endogenous Argonaute-bound RNAs (CLEAR)-CLIP^10^ experiments on MDBK cells (Supplementary Table S1). Experiments with cells infected with bovine viral diarrhea virus (BVDV), which sequesters miR-17^14^, or cells treated with miR-17 inhibitor were included for identification of interactions. These were however excluded in analyses of individual miRNA profiles since these manipulations led to underrepresentation of miR-17 interactions (Supplementary Table S1). From the 39 data sets, more than 20 million AGO-bound reads uniquely mapped to the cow genome and clustered into ~3.8 million regions, of which ~1.4 million were identified in >1 biological replicate (i.e. biological complexity [BC] > 1). Analysis of peaks significantly enriched over background identified ~240,000 AGO binding sites (Supplementary Table S2).

**Figure 1:**
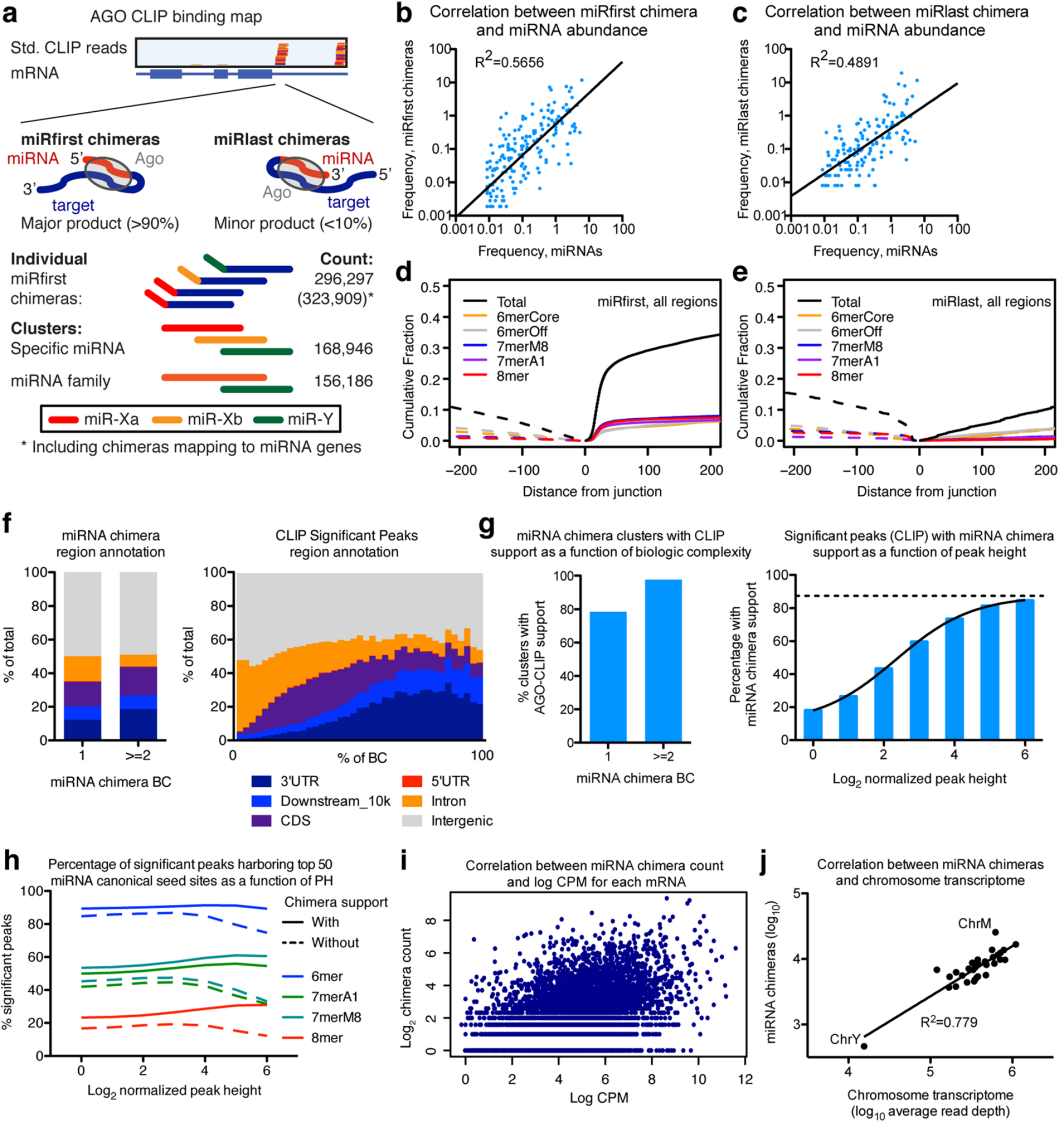
Identification and characterization of miRNA-target chimeras. **(a)** Schematic representation of AGO-CLIP reads mapping to an mRNA and of the two types of miRNA-target chimeras, miRfirst and miRlast (top). Schematics and numbers of individual chimeras, clusters, and family clusters (bottom). **(b**,**c)** Correlation between frequencies of AGO-loaded miRNA-chimeras and individual miRNAs for miRfirst (**b**) and miRlast (**c**) chimera types. **(d**,**e)** Cumulative plots showing enrichment for canonical miRNA seed sites upstream and downstream in target sequences relative to the chimeric junction site. **(f)** Genomic region annotation for miRNA chimeras (left) or for significant CLIP peaks (right) as a function of BC. **(g)** Overlap between miRNA chimera and standard CLIP data shown as a function of miRNA chimera BC (left) or as a function of normalized height for significant CLIP peaks (right). **(h)** Percentage of significant peaks harboring top 50 miRNA canonical seed sites as a function of peak height (PH) is shown for chimera supported and non-supported peaks. **(i)** Correlation between miRNA chimera count and log(CPM) for each mRNA. **(j)** Correlation between miRNA chimera count and chromosome transcriptome size.

Using our recently established approach to unambiguously identify miRNA-target interactions^10^, we identified ~300,000 miRNA-target chimeras (Fig. 1a). We observed a good correlation between miRNA abundance and abundance of miRNAs in chimeras both for the miRfirst and miRlast type chimeras (Fig. 1b,c). In human and mouse less correlation was observed for miRlast^10^, presumably due to spuriously annotated human- or mouse-specific miRNAs affecting this lower saturated data set. Consistent with our prior analyses, strong enrichment for canonical miRNA seed sites was found for miRfirst chimeras, in particular on 3′ UTRs (Fig. 1d and Supplementary Fig. S1a–d), whereas less enrichment was observed for miRlast chimeras (Fig. 1e and Supplementary Fig. S1e,f). Thus, further analysis was focused on miRfirst chimeras (henceforth “chimeras”; Supplementary Table S3), which constituted 0.5–5.0% of the total uniquely mapped reads. Clustering of target regions bound to specific miRNAs identified ~170,000 unique miRNA-target interactions (~1.8-fold collapsing), and seed site enrichment increased with the cluster size (Supplementary Fig. S1g–i). This dataset is thereby the deepest reported to date.

Region annotation of chimeras was similar to that of standard AGO-CLIP data (Fig. 1f and Supplementary Fig. S1j). Among unique miRNA-target interactions, ~98% of chimeras present in at least two datasets overlapped with standard AGO-CLIP reads. Conversely, the percentage of significant CLIP peaks supported by chimeras increased as a function of the normalized peak height. Sigmoidal regression suggested a maximum possible 88% chimera supported CLIP clusters (Fig. 1g and Supplementary Fig. S1k). The seed site frequency for the 50 most abundant miRNAs declined for the most robustly defined significant peaks without chimera support but not for those with chimera support (Fig. 1h). This suggested that a fraction of standard AGO-CLIP peaks may not be miRNA associated. However, a certain bias towards identifying chimeras at bona fide sites e.g. due to steric hindrance can not be excluded. Further, the number of miRNA chimeras scaled with the log(CPM) values of the individual mRNAs and the overall transcriptome size per chromosome, as evaluated by mRNA-seq (Fig. 1i,j). Taken together, chimera-defined and AGO-CLIP-defined sites appeared similar, and the greater depth of this data set could allow analyses not previously possible.

### Novel annotations of bovine miRNAs

By analyzing AGO-bound miRNAs in MDBK cells, we found the abundance profile dominated by members of the miR-30, let-7, miR-17, miR-374, miR-21, miR-27 and miR-15 families together accounting for more than 50% (Supplementary Fig. S2a)^14^. After mapping to mature miRNAs annotated in miRbase^17^, we further analyzed unmapped 17–25nt sequences. This identified non-annotated 5p or 3p variants of four miRNAs; miR-224-3p, miR-324-3p, miR-340-5p and miR-542-3p (Table 1 and Supplementary Fig. S2b). These constitute novel additions to bovine miRNA annotation, and demonstrate the usefulness of our method in identifying novel miRNAs that engage in functional interactions.

**Table 1:** Suggested updates to bovine miRNA definitions from this data set.

miRNA	Current annotation	Sequence from this data set	Sequence count
bta-miR-224-3p	Only 5p annotated	CAAAATGGTACCCTAGTGACT	443
bta-miR-324-3p	Only 5p annotated	CCACTGCCCCAGGTGCTGCTGG	652
bta-miR-340-5p	Only 3p annotated	TTATAAAGCAATGAGACTGA	669
bta-miR-542-3p	Only 5p annotated	TGTGACAGATTGATAACTGAA	475

For bta-miR-340-5p and bta-miR-542-3p, despite lack of annotation, a deep sequencing profile consistent with what is reported here is presented in miRbase.

### Overlap of identified miRNA-target interactions with validated targets and predictions

**Figure 2:**
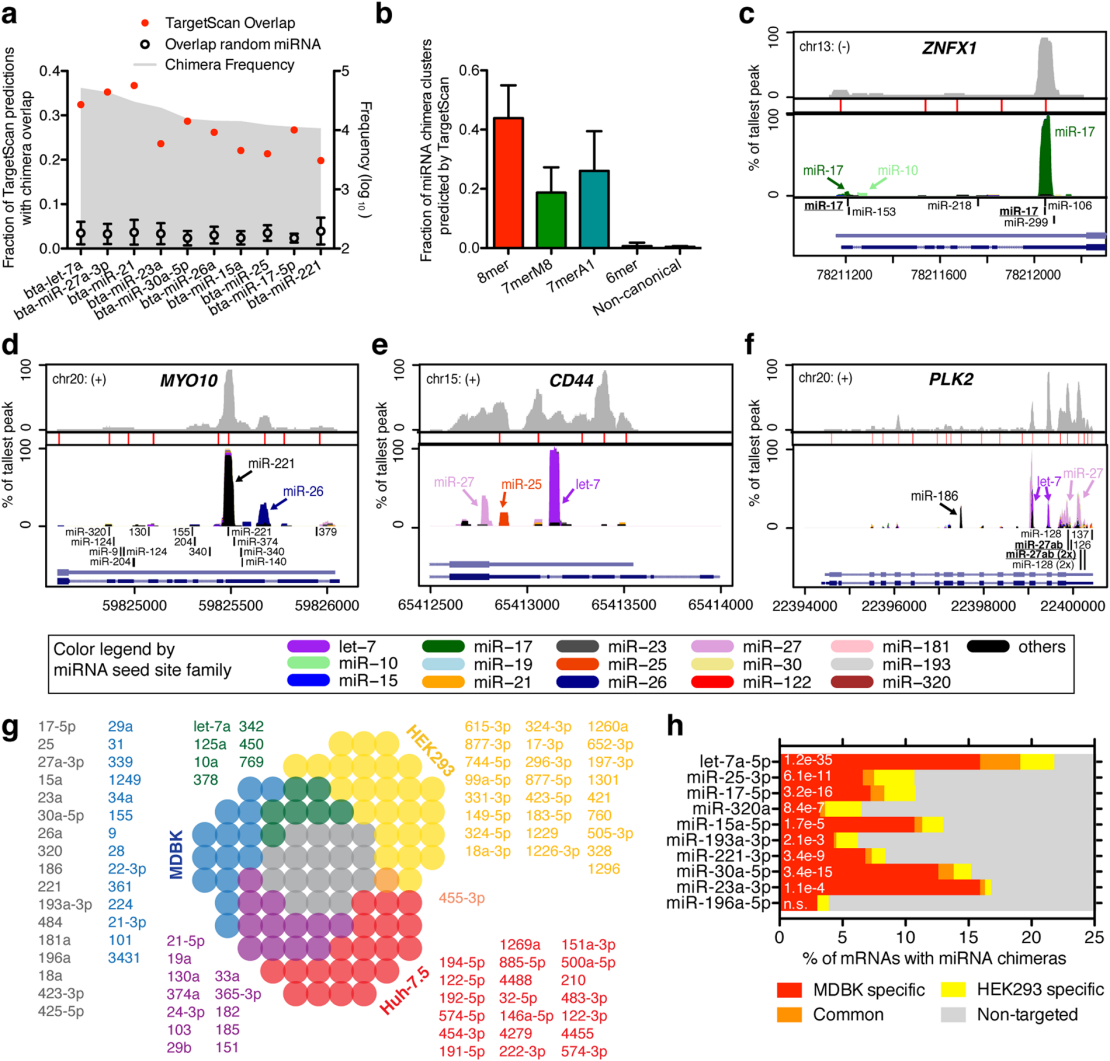
miRNA chimera defined target sites and overlap with TargetScan. **(a)** Fraction of TargetScan predictions with chimera overlap shown for the most abundant miRNAs. **(b)** Fraction of miRNA family chimera clusters predicted by TargetScan, shown by presence of canonical seed site. **(c**–**e)** Specific mRNA 3′ UTR profiles of AGO-CLIP data (top, light grey), significant peaks (red bars), miRNA chimera data (middle, colored by miRNA family, see legend at the bottom), TargetScan predictions (bottom, black bars) and mRNA structure (bottom, light blue: cow; dark blue: human/others). **(f)** miRNA target data as in (**b**–**e**) but for the entire *PLK2* mRNA. **(g)** Overlap of top 50 miRNAs between bovine kidney (MDBK), human kidney (HEK293) and human liver (Huh-7.5) cells is shown as a to-scale Venn diagram. Color coded miRNAs from each group are listed in order of abundance. **(h)** Overlap in miRNA targetome between MDBK and HEK293 cells is shown as percentage of chimera targeted mRNAs for individual miRNAs. The significance of overlap was evaluated using the hypergeometric test, and p-values are given. n.s.: not significant.

To validate captured sites with existing support and enumerate novel ones, we compared CLIP-defined sites to established databases and prediction tools. According to miRTarBase, only two miRNA-target interactions have been experimentally validated in the cow, whereas another ~100 targets miRNA interactions were only inferred by changes in RNA abundance^18^. Thus, our data tremendously expand the number of validated interactions. TargetScan is a prediction algorithm for miRNA interactions in 3′ UTRs considering sequence context and evolutionary conservation^5^. Evaluation of broadly conserved TargetScan predictions (present in both human and cow) showed that our experimental approach agreed with up to 40% of these, demonstrating partial overlap with bioinformatic predictions (Fig. 2a). As expected, less overlap was observed for less abundant miRNAs presumably due to sub-saturating capture of chimeras. Similarly, TargetScan predicted ~40% of chimeras harboring 8mer seed sites on 3′UTRs, but only ~25% of 7mers and almost none of 6mers and non-canonical interactions (Fig. 2b). Examples of genes for which the largest miRNA chimera peaks were predicted by TargetScan include *ZNFX1*, *ABCA1*, *THBS1*, *CDKN1A* and *WEE1* (Fig. 2c and Supplementary Fig. S2c–f), partial prediction was observed e.g. for *ANXA2* and *MYO10* (Fig. 2d and Supplementary Fig. S2g), whereas prediction failed in other cases such as *CD44* (Fig. 2E). In addition, miRNA chimeras revealed peaks in other genic regions not assessed by TargetScan (e.g. *PLK2*, Fig. 2f). Thus, the experimental approach proved superior, in particular for finding miRNA interactions not supported by strong canonical seed sites or in regions other than 3′UTRs.

The miRNA interactome in HEK293 human kidney cells was previously reported^8^, and we therefore analyzed the overlap with bovine kidney cells. Among chimeras of the 50 most abundant miRNAs, HEK293s as expected had more miRNAs common with bovine kidney (MDBK) compared to human liver (Huh-7.5 cells^10^, Fig. 2g). MDBK cells, however, shared slightly more miRNAs with Huh-7.5 s compared to HEK293s. This might be explained by the different developmental stage of MDBK/Huh-7.5 (adult) vs. HEK293 (embryonic), modifications done to HEK293 cells^8^, or methodological bias for HEK293s (CLASH) compared to MDBKs and Huh7.5 s (CLEAR-CLIP). Several spurious miRNAs with highly repetitive sequences (e.g. miR-744-5p, miR-877-3p, miR-1229) were present only in the CLASH data set, and such false positives would reduce the overlap. For the 10 miRNAs each present in >=1% of both the MDBK and HEK293 chimeras, hypergeometric test showed significant overlap between targeted mRNAs for 9 out of 10 (Fig. 2h). Overall this demonstrated a shared miRNA interactome between human and bovine kidney cells.

A single study previously performed AGO-CLIP on bovine tissue^19^. Through analysis of overlap with chimeras, the miRNA responsible for binding at 149 of 348 (43%) significant CLIP peaks from that study on bovine retina were putatively identified (Supplementary Table S4). Of these, 47 (13.5% of total) were robust with at least three chimeras of the given seed family supporting the interaction. Thus, despite tissue and miRNA repertoire differences, miRNA chimera data were useful for uncovering specific interactions.

### miRNA chimeras predict transcriptome changes induced by miR-17 sequestration

**Figure 3:**
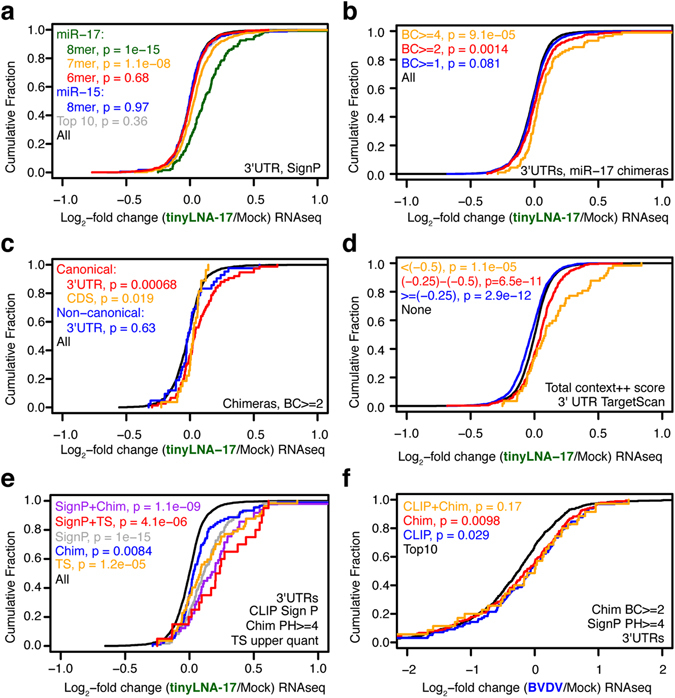
Comparison of experimental and bioinformatic predictors of miR-17 regulation. **(a**–**e)** Cumulative density function (CDF) of the log_2_-fold change in mRNA abundance between tinyLNA-17 and mock treated MDBK cells is shown using grouping of mRNAs by (**a**) presence of miRNA seed sites in 3′ UTR significant AGO-CLIP peaks, (**b**) BC of miR-17 chimeras in 3′ UTRs, (**c**) canonical or non-canonical miR-17 chimeras in CDS and 3′ UTRs, (**d**) 3′ UTR TargetScan7 context++ score for miR-17, or (**e**) combining the experimental and bioinformatic predictors. **(f)** CDF plots of the log_2_-fold change in mRNA abundance between BVDV(cp) infected and mock treated MDBK cells grouping mRNAs by presence of significant AGO-CLIP peaks or miR-17 chimeras on 3′ UTRs. Two-sided K-S test p-values are shown.

To evaluate how accurately miRNA chimeras predict valid regulatory miRNA interactions, we performed mRNA-seq on MDBK cells treated with the miR-17 family (miR-17, -20, -93 and -106) inhibitor, tinyLNA-17, and analyzed changes in steady state RNA levels across the transcriptome (Supplementary Table S5). The presence of a miR-17 7- or 8mer, but not 6mer, seed site within significant CLIP peaks was robustly predictive for mRNA de-repression, in particular if the seed was present in 3′ UTRs (Fig. 3a and Supplementary Fig. S3a-c). For 8mers, the median log_2_ fold change of was 0.11. Similarly, the presence of miR-17 chimeras was predictive for regulation with a median log_2_ fold change of of 0.03 (Fig. 3b and Supplementary Fig. S3d). Chimeras representing canonical miR-17 interactions were predictive both on 3′ UTRs and coding sequence (CDS); whereas there was a trend for non-canonical chimeras this was not statistically significant (Fig. 3c). Thus, as previously observed^10^, miRNA chimeras as well as the combination of significant CLIP peaks and strong canonical seed sites predicted miRNA regulation, even outside 3′ UTRs. As previously shown for other miRNAs, simply predicting miR-17 targets using TargetScan identified de-repressed mRNAs, although only mRNAs with context++ scores <(−0.25) were significantly de-repressed (Fig. 3d). The lowest scoring mRNAs <(−0.5) had median log_2_ fold change of 0.09. Finally, combining predictors markedly increased prediction of de-repression with median log_2_ fold changes of 0.17 and 0.22, respectively, for significant peaks combined with the presence of miR-17 chimeras or TargetScan predictions (Fig. 3e). However, this further narrowed the gene set and thereby the statistical power.

BVDV specifically sequestrates miR-17, and we previously showed that robust infection leads to a functional de-repression of mRNAs with miR-17 8mer related AGO-CLIP peaks^14^. Here, using miRNA chimeras to predict BVDV miR-17 “sponge” regulated genes we confirmed the finding with even higher statistical significance. Combining presence of CLIP peaks and miR-17 chimeras led to a marginally increased prediction (Fig. 3f).

We conclude that experimental approaches using miRNA chimeras enhanced identification of valid regulatory miRNA sites, particularly when combined with other indicators, such as standard AGO-CLIP data.

### miRNA chimeras identify known ceRNAs

Competing endogenous RNAs (ceRNAs) have been proposed to regulate the miRNA pool as opposed to being regulated by miRNAs themselves^3^. ceRNAs were previously reported to regulate several miRNAs expressed in MDBK cells^20^. Among protein coding ceRNA-miRNA pairs, we confirmed strong interactions for HMGA2/let-7, KRAS/let-7 and the PTEN network although abundant binding of other miRNAs was also evident for some of these RNAs. The interaction between let-7 and the non-coding H19 RNA was also confirmed (Table 2). To identify novel putative bovine ceRNAs, we identified regions with at least three clusters each consisting of at least five unique chimeras within a genomic region of 3000 bp. However, most such regions fell within annotated genes and mostly in 3′UTRs (Supplementary Table S6). These therefore appeared to be examples of classical miRNA repression, rather than ceRNA type miRNA sponges. We conclude that miRNA chimeras identified known ceRNAs, whereas no suspected novel ceRNAs were identified in MDBK cells.

**Table 2:** Previously described ceRNAs relating to the 50 most abundant miRNAs in MDBK cells.

Type	Gene	ceRNA	Ann cow	miRNA	MDBK 3′UTR miRNA chimeras
Non-coding		H19	Yes	let-7	let-7
Coding	PTEN	PTENP1	No	miR-21	Various miRNAs, mostly miR-27
		CNOT6L	Yes	miR-17, miR-19	miR-17 and miR-25
		VAPA	Yes	miR-17, miR-19	miR-17
		ZEB2	Yes	miR-26, miR-25	None
	KRAS	KRAS1P	Yes	let-7	let-7 present, but mostly miR-27 and miR-222
	HMGA2		No	let-7	let-7
		TGFBR3	No	let-7	let-7 peak in exon, miR-15, -17, -21, -27 and -31 peaks in 3′UTR

Definitions of previously described ceRNAs in human or mouse were obtained from ref. 20.

### miRNA-target de novo motif prediction and interaction analysis identifies pairing patterns

**Figure 4:**
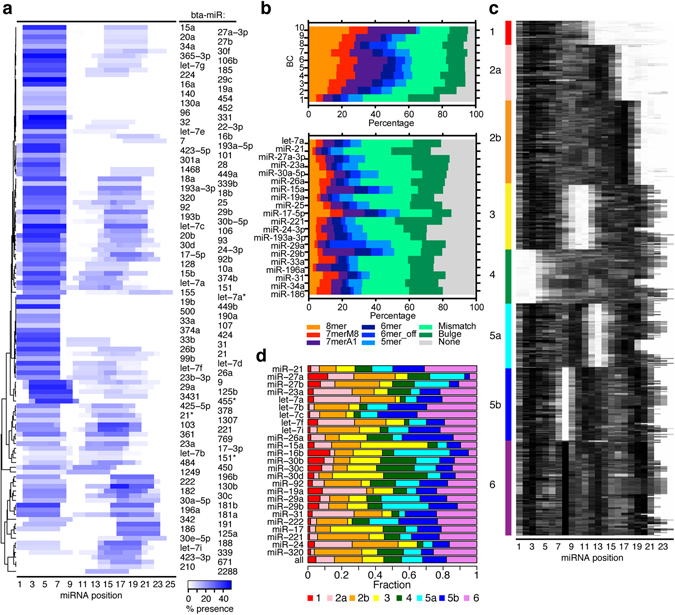
miRNA pairing rules for bovine miRNAs. **(a)** De novo analysis of cognate miRNA-complementary-enriched 7mer motifs in chimera target regions plotted as a heat map across the miRNA. miRNAs are ordered by hierarchical clustering. Coloring is proportional to percent-wise motif presence. **(b)** The proportion of chimera-defined target regions with the indicated seed variants is plotted, broken down by chimera BC (top) or the most abundant miRNAs (bottom). **(c)** miRNA–target duplex structure predictions represented as heat maps. Black pixels indicate base pairing. Structures were partitioned by k-means clustering into eight groups. Interactions from all transcript regions were included in this analysis. **(d)** Distribution of the identified clusters for the most abundant miRNAs. miRNAs were grouped by seed site family and ranked by decreasing abundance.

Unbiased de novo motif (7mer) analysis of chimera target regions identified strong enrichment of seed-complementary motifs, albeit with great variations among individual miRNAs (Fig. 4a). In addition, motifs representing auxiliary pairing were evident for most miRNAs peaking at positions 14–20. Among miRNAs without significant seed binding were miR-30e and -125a, both from families for which we previously demonstrated the importance of non-canonical binding^10^. Yet, for the human and murine orthologs of these miRNAs, seed pairing was observed. Absence of significant seed pairing may also be explained by the lower sampling (<100 chimeras) of some miRNAs in this group. We observed overlap when comparing de novo motif prediction for bovine miRNAs to previous analyses for human liver cells and mouse brain^10^, although differences were observed in auxiliary pairing (Supplementary Fig. S4a,b). For example, auxiliary pairing was more present for members of the miR-30 family in the cow compared to the mouse, whereas the opposite was true for members of the miR-19 family. This suggests a conserved seed site usage but a variable auxiliary pairing usage between species. Sub-saturating coverage, however, may alternatively explain these differences.

To identify miRNA-target pairing patterns for individual binding sites, we predicted the most favorable binding energy for each miRNA-target interaction using RNAhybrid (Supplementary Table S7)^21^. This allowed identification of mismatched, bulged and 3′ pairing in addition to canonical seed sites. On 3′ UTRs, more than 80% of interactions fell into one of these categories, and the percentage increased with BC and peak height (Fig. 4b and Supplementary Fig. S4c). Large variation in preference for interaction type was observed between different miRNA families (Fig. 4b). k-means clustering of structures reflected the previously identified six major modes of miRNA–target binding^10^, with five dominated by seed site pairing combined with various auxiliary binding patterns (Fig. 4c). Two previously identified groups, that of seed pairing with loosely connected auxiliary pairing (group 2) and that with bipartite auxiliary pairing (group 5) could be further divided into two sub-populations in this deeper data set, based on the precise sites engaged in auxiliary base pairing. As observed previously, individual miRNAs showed a wide range of binding site preferences with many tolerating two or more structures (Fig. 4d). Thus, the nature of miRNA-target pairing in bovine cells overall reflected that previously observed in human and mouse.

### Gene ontology reveals function of gene sets regulated by specific miRNAs

**Figure 5:**
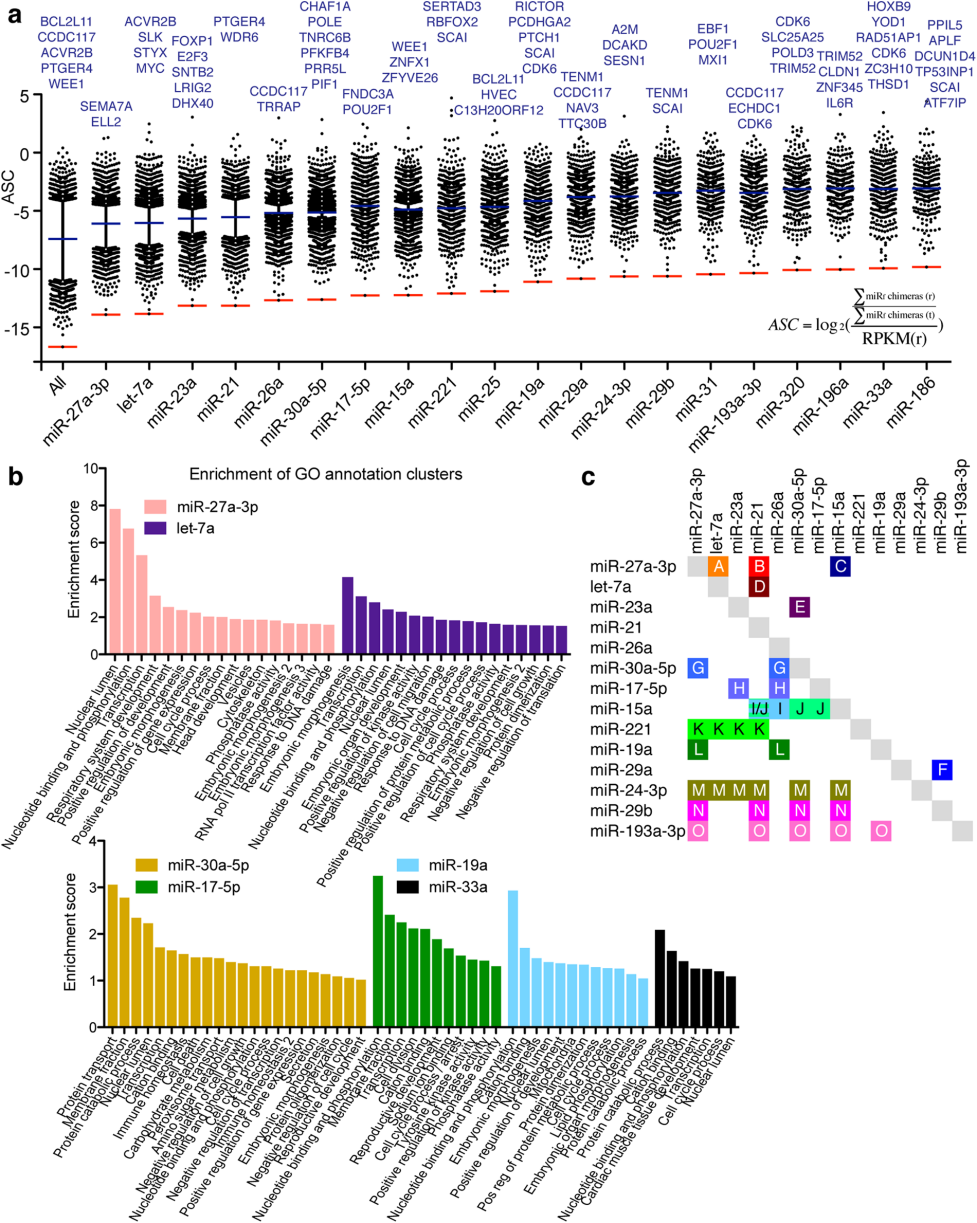
Gene targeting profile for the most abundant miRNAs. **(a)** ASC values are plotted for the most abundant miRNA families. The most highly targeted mRNAs are noted. Blue lines indicate mean values (excluding non-targeted mRNAs); red lines indicate lower level of quantification. In the calculation of ASC values, f denotes the miRNA family of interest, r the mRNA in question and t total number. **(b)** GO analysis on gene sets with high ASC values for selected miRNA families. Enrichment scores of annotation clusters are shown. **(c)** Cooperativity of miRNA families in mRNA targeting. Pairwise (upper right) or multi-family (lower left) miRNA family targeting cooperatives are highlighted. Color-coding and letter designation refers to k-means clusters of genes by miRNA specific ASC values (refer to Figure S5b–d). Highlighted groups occurred in all three iterations of analysis, except for groups C, I and K that occurred in only two.

We previously defined the AGO sequestration coefficient (ASC) as an RNA abundance normalized measure for total AGO sequestration by specific RNAs^14^. To define gene sets regulated by specific miRNAs, we extended this to miRNA-specific parameters, defined as the log_2_ transformed number of 3′ UTR bound miRNA family chimeras normalized by the RPKM value for the given mRNA. miRNA families with >500 chimeras were included (Fig. 5a and Supplementary Fig. S5a and Table S8). This revealed the mRNAs most highly targeted by specific miRNAs. We next performed gene ontology (GO) analysis of mRNAs with the highest ASC for selected miRNAs (Fig. 5b). This confirmed a number of known functions e.g. of the let-7 family in suppressing embryonic development and cell cycle progress^22, 23^, and the miR-17 family in suppressing cell cycle arrest^24, 25^. GO analysis also revealed potentially novel regulation, such as that of miR-27 in transcriptional and membrane/vesicle-associated processes, miR-30 in immune homeostasis and cellular metabolism and transport, and miR-33 in cardiac development.

To understand cooperative regulation between different miRNAs, we clustered mRNAs by miRNA-specific ASC using k-means analysis. From three iterations, each from 100,000 random starts (Supplementary Fig. S5b-d), consistent patterns of miRNA cooperativity were identified (Fig. 5c). As expected, the most abundant miRNA families engaged in most combinations. Examples of cooperativity among abundant miRNA families included all pairwise combinations of let-7, miR-21 and -27. We also identified several preferred multicomponent interactions, including those of miR-15, -21, -27 and -30. Interestingly, miRNA families encoded by the same cistron, such as miR-23, -24 and -27 or miR-17, -19 and -25, did not preferentially cooperate. As a positive control of ASC scores and cooperativity, we confirmed that miR-29a and -29b, which share the same seed sequence, clustered strongly in this analysis.

Thus, miRNA chimeras allowed establishment of a detailed weighed interactions map from which GO and cooperative information could be deduced. These findings should solidly enhance understanding of bovine miRNA regulation and act as a significant resource for future studies.

## Discussion

Despite significant improvement in miRNA-target predictions, experimental approaches remain essential in delineating binding landscapes across transcriptomes. With ~170,000 interactions identified, the current analysis of 39 AGO-CLIP and CLEAR-CLIP data sets provides the most comprehensive experimental mapping of miRNA interactions in any species so far. As such, it encompasses a significant resource for the community, and in particular for studies of molecular biology in cattle. Other than a handful of experimentally validated targets, only correlative predictions were previously available in cattle^18^. Thus, the current study expands the number tremendously.

Our approach proved to be powerful in identifying miRNA targets. The only ~40% overlap with TargetScan predictions on one hand reflects predicted sites that may not be used in the cell type in question but also the continued sub-saturation of experimentally derived data sets. On the other hand, the current data set further identifies canonical 6mers and non-canonical interactions, functional interactions outside of 3′ UTRs, as well as species-specific interactions, which are typically not identified by prediction algorithms such as TargetScan. miRNA-target chimeras also significantly predicted functional miR-17 regulation, although the most conservative bioinformatic parameters, such as the presence of 8mer seed sites or the highest TargetScan scores, were better predictors of the most highly regulated genes. This may come as no surprise, given that miRNA-target chimeras in addition to functionally regulated sites would also identify other binding sites such as those buffering the available miRNA pool. Such sites would include those competing with functional regulation according to the ceRNA model^3, 26^. Nonetheless, the most powerful prediction of regulatory miR-17 sites came from combining experimental and bioinformatic approaches (Fig. 3). Further emphasizing the combined value of experimental and predictive approaches, data such as that of the current study could be integrated in training of prediction tools, thereby improving these for the future^27^.

Experimentally validated miRNA-target interactions allowed deeper investigation of miRNA pairing rules in cattle. Using de novo motif and RNAhybrid predictions we identified patterns similar to what was previously reported in human and mouse^8, 10^. De novo motif prediction confirmed the importance of canonical seed sites for the vast majority of miRNAs. Interestingly, significant seed binding was not found for miR-30e and -125a, both from families for which we previously demonstrated the importance of non-canonical binding^10^. Lack of enriched seed pairing, however, might also be a result of lower miRNA abundance, and thus limited sampling. Comparison with human and mouse further suggested conserved seed site usage but variable auxiliary pairing usage between species. Individual chimera predictions using RNAhybrid, in particular for targets confirmed by multiple biological replicates, showed that >90% had some sort of canonical interactions when 5mer motifs, mismatches and bulges were allowed. For example, we previously showed that 8mer bulge interactions can be functional^10^. When clustering pairing patterns across bovine miRNAs by k-means analysis, 8 groups were identified. These reflected those previously identified in human and mouse^8, 10^, including seed-only pairing (1), seed with auxiliary pairing (2–3), and seed with bi-partite (5) or tri-partite (6) auxiliary pairing. A group without seed pairing was identified (4), whereas a group lacking significant miRNA–target pairing identified by CLASH^8^ was not seen here. From the deep bovine data set, two groups (2 and 5) could be further divided (2a/b, 5a/b) depending on the position of auxiliary pairing. The latter (5a/b) was similar to what was previously found in Huh-7 derived human hepatoma cells^10^. In addition to the deeper data set, this cluster diversity may reflect the diversity in the MDBK miRNA profile, which included many miRNAs expressed at high to moderate levels (Supplementary Fig. S2).

In addition to target identification, AGO-CLIP proved useful in identifying AGO loaded miRNAs. Compared to general small RNA sequencing, this adds an additional functional level to see what miRNA population is actually engaged in the RISC complex. Here, we used this to define the bovine miRNAs, miR-224-3p, miR-324-3p, miR-340-5p and miR-542-3p, not previously registered in miRBase^17^.

The relatively deep probing of the miRNA-target landscape allowed interrogation of target networks of specific miRNAs. The let-7 family is known to suppress embryonic development and cell cycle progression^22, 23^ and to play a role in DNA damage response^28, 29^. These regulatory functions were confirmed by our GO analysis that also suggested unappreciated roles in transcription and translation. The miR-17 family, and to some extent also miR-19, in contrast suppresses cell cycle arrest and there is some evidence for targeting kinases like MAPK14^24, 25^. These regulatory themes were confirmed by our GO analysis. Due to expression of miR-17 and -20 from the miR-17~92 cluster that also contains miR-18, -19 and -92a, and the parallel expression of the other family members miR-93 and -106 from other clusters, careful delineation of the function of individual families and family members remains to be done. A number of roles have been suggested for miR-27, which is expressed in a cluster with miR-23 and -24^30^. Roles confirmed by our GO analysis included differentiation of embryonic stems cells^31^ (“positive regulation of development”) and the DNA damage response^32^. Potentially unappreciated regulation included transcriptional and membrane/vesicle associated processes. Although several roles have been suggested for the miR-30 family, including regulation of adipogenesis, lipid metabolism, cardiovascular disease and cancer^33^, this family remains inadequately characterized. Interestingly, our analysis further suggested roles in immune homeostasis and cellular metabolism and transport. While our analysis did not identify the known involvement of miR-33 in lipid metabolism^34^, a novel potential role in cardiac development was suggested. Thus, our use of miRNA-target chimeras in analysis of gene sets regulated by specific miRNAs proved useful for confirming a number of known roles as well as suggesting putative unappreciated roles in regulation.

In conclusion, the current study presents the most comprehensive miRNA binding landscape in any species reported to date. With its higher depth, it demonstrates that experimental mapping of miRNA interactions at saturating levels is within reach. Unambiguously identified miRNA targets will be useful for research on regulation of particular genes or miRNAs. In addition, this approach proved useful for identifying GO pathways affected by particular miRNAs and for studying cooperative behavior between miRNA families. The resulting datasets provide a significant resource for the miRNA field.

## Experimental Procedures

### Cell culture

MDBK cells were cultured in DMEM with 10% horse serum, non-essential amino acids and 0.1 mM sodium pyruvate. For tinyLNA-17 (AGCACTTT, all LNA, Exiqon) treatment, lipofectamine RNAi/MAX (Life Technologies) was mixed with LNA (300 nM final concentration) in Opti-MEM and incubated for 5 min. The solution was then added on top of the monolayer growing in fresh media and incubated for 24 hrs before RNA extraction.

### CLIP and RNA-seq library preparation

Standard AGO-CLIP was done as described^35^. To enable cost-effective multiplexing, we added sequencing adapters and 5′ indices in the 2^nd^ PCR step using the primers listed in Table S6 of ref. 14. This strategy uses the DP5 and DP3 sequences of the 1^st^ PCR product as priming sites to add 5′ indices and 3′ adapters for a short (6–16 cycles) 2^nd^ PCR step.

CLEAR-CLIP is based on standard AGO-CLIP with modifications to enrich for miRNA-target chimeras^10^. Briefly, the following procedures replace the post-immunoprecipitation steps of standard AGO-CLIP: (i) 5′-end phosphorylation using PNK (3′ phosphatase minus), (ii) Over-night chimera ligation using T4 RNA ligase 1, (iii) Alkaline phosphatase treatment to remove 3′ phosphate groups, (iv) 3′-linker ligation using truncated T4 RNA ligase 2 and pre-adenylated linker (using a pre-adenylated linker and omitting the enzyme in step ii allows negative controls to distinguish cellular vs. on-bead ligation events), and (v) Radio labeling using T4 PNK and [γ-^32^P]-ATP. Here, the ligase-free controls used to establish the method^10^ were not needed, and the 3′ linker ligation was therefore done with T4 RNA ligase 1 and a radioactively labeled phosphorylated RNA linker according to the standard AGO-CLIP protocol^35^, and not using truncated T4 RNA ligase 2 and a pre-adenylated linker. Accordingly, the subsequent PNK treatment was done with non-radioactive ATP.

The mRNA-seq libraries were prepared from Trizol extracted RNA following Illumina TruSeq protocols for poly-A selection, fragmentation, and adaptor ligation. Libraries were sequenced as 100 bp single-end runs (Illumina).

### Bioinformatic analyses

#### Mapping of AGO-CLIP data

AGO-CLIP data was processed as described^35, 36^ and aligned to the bovine genome (BosTau7) using Bowtie^37^. After mapping, reads were collapsed on coordinates such that only those with sufficiently different degenerate linkers were kept; this distinguished unique binding events from PCR duplicates^35^. AGO-CLIP alignment statistics are given in Supplementary Table S1. Significant peak analysis was done using the findPeaks tool of the Homer package^38^ using a fragment length of 53 nts (based on evaluation of mean fragment size by the makeTagDirectory tool) and a minimum inter-peak distance of 75 nts.

AGO-bound miRNA profiles were deduced by bowtie aligning to mature bovine miRNAs (miRbase, downloaded on 14 May 2014) allowing for zero mismatches.

#### Identification of miRNA-mRNA chimeras

Sequence reads containing miRNA sequences were identified by reverse mapping mature miRNA sequences against sample libraries using Bowtie. Changes to default parameters were: 5′ trimming (−5 = 1), maximum mismatches allowed in the seed (−n = 1); seed length (−l = 18); maximum total of quality values at mismatched read positions (−e = 30). This allowed mapping of miRNAs with a maximum of 1 mutation in the first 18 nts and 5′ or 3′ truncation of 1 nt. Sequences upstream and/or downstream of miRNAs within reads were extracted in “R” using the coordinates supplied by Bowtie. For reads with similar miRNAs mapping within 4 nts, the first alphanumeric miRNA was chosen such that only one event was represented. Reads with multiple mature miRNAs mapping to different coordinates within the read (such as pre-miRNA chimeras or miRNA-miRNA chimeras) were allowed. Chimeras of miRNAs not present in MDBK cells were excluded, including certain spurious miRNAs, such as miR-2476, which appears to be a tRNA fragment (miRbase). Target sequences >=19 nts were then mapped to the bovine genome using bowtie. PCR duplicates were consolidated on the basis of genomic coordinates and a degenerate barcode present in the 5′ RNA linker. Chimeric fragments mapping to miRNA precursor genes were removed and miRNA family nomenclature was added based on the 8mer seed site.

#### Analysis of AGO-CLIP and miRNA chimera data

In general, data analysis was done in R using GenomicRanges, Biostrings, genomeIntervals, Hmisc and gplots packages^39^. Annotation (genic region, gene ID, RMSK) was done by intersection with BosTau7 GTF files (UCSC table browser). Due to incomplete annotation of the bovine genome, the 10k region downstream of 3′ UTRs was included in analyses of 3′ UTRs. To enable extraction of target sequences in R, BSgenome.Btaurus.UCSC.bosTau7 was created from bosTau7.2 bit (UCSC) using BSgenomeForge. Custom colored bedgraphs were visualized in R using the *plot.stacked.r* function (http://menugget.blogspot.dk/2013/12/data-mountains-and-streams-stacked-area.html).

#### Analysis of changes to the host transcriptome

Quality filtered RNA-seq data were mapped to the bovine genome (bosTau7) using Bowtie. The number of reads overlapping each gene (UCSC and Ensembl annotation) was counted with htseq-count^40^. Expression data was processed using edgeR^41^. RNA-seq derived gene expression data was merged on gene name with AGO-CLIP data to allow CLIP-guided representation of changes to gene expression.

#### Intersection with TargetScan

To intersect data with TargetScan predictions^5^, predicted sites were downloaded from the human hg19 UCSC table browser, and coordinates were lifted to the BosTau7 genome (>0.1 ratio of conserved bases; this kept 53,455 of 54,199 interactions). This strategy allowed intersection of “broadly conserved” sites present in both human and cow. Genes not expressed in MDBK cells (<100 tags in RNA-seq) were excluded from analysis. Intersection was done using the genomeIntervals package in R.

#### Intersection of miRNA gene regulation with other data sets

The top 50 most abundant miRNAs present in chimeras were inferred from previously published data sets of modified HEK293 human kidney cells^8^ and Huh-7.5 human hepatoma cells^10^. Differences in human and bovine miRNA nomenclature was handled manually. For evaluating targeting overlap, miRNAs present in >=1% of chimeras for both MDBKs and HEK293s were selected and gene overlap was assessed using the hypergeometric test. Only mRNAs for which at least one chimera was observed in any genic region in both data sets were included to consider CLIP sensitivity.

#### Motif analysis

For de novo motif analysis of chimera target sequences (75 nts), chimeras were grouped for each miRNA present in at least 50 individual chimeras, representing at least 40 individual clusters. Using R, foreground (target) and background sequences were written to fasta files. Background sequences totaling 5 times the number of foreground sequences were randomly selected from other miRNA chimeras, excluding other miRNAs with identical canonical seed sites. De novo motif discovery was performed on triplicates of these sequence sets using Homer, expecting 7mer motifs and checking motifs for complementarity to the cognate miRNA, using commands similar to:

perl bin/findMotifs.pl foreground/bta-miR-17-5p.txt fasta output/bta-miR-17-5p/ -fasta background/bta-miR-17-5p.txt -mcheck motifs/bta-miR-17-5p.motif -norevopp -noknown -len 7 –bits.

To this end, reverse complement miRNA sequences were added to the Homer list of known motifs using commands similar to:

perl bin/seq. 2profile.pl ACTACCTGCACTGTAAGCACTTTA 0 bta-miR-17-5p> motifs/bta-miR-17-5p.motif.

Information from Homer output files was extracted using regular expressions in R, and a combined confidence parameter, c, was calculated as

$${\rm{c}}=(-\mathrm{log}\,10({\rm{p}})-10)/10+({\rm{s}}-0.35)\,\ast \,6.7,$$

where p is the p-value and s is the match score with the given miRNA as outputted by Homer. Only motifs with s >=0.35, information content per bp >=1.75 and c >=1 were considered for further analysis. This allowed only motifs with matching 5/7 positions (down to 5mers), non-degenerate motifs, and motifs meeting the confidence threshold of combined significance and match to the miRNA. In iterations of random comparisons of background sequences, p-values below 1e-10 were rarely observed, and c-values meeting the threshold were never observed. Heat-maps were created in R using the gplots package.

#### RNA duplex structure prediction

For each chimera cluster, duplex structure predictions were made on 75 nt target regions with the cognate miRNA using RNAhybrid^10, 21^. Clusters >100 nts in length were omitted, as an accurate structure prediction was deemed unlikely in a large interval. Clusters >75 nts and =<100 nts were trimmed symmetrically from both ends to a length of 75 nt. We reasoned that canonical seed matches and variants were likely to be engaged in base pairing when present. To improve concordance with motif presence, pairing was forced in appropriate seed positions when perfect 8mer, 7mer, 6mer, or 5mer matches were present. This modification, along with adjustments of allowed loop/bulge size (−u = 15; –v = 5), improved concordance for canonical sites. For targets with mismatch (8mer, 7mer, and 6mer) or bulge (8mer and 7mer) motifs, two duplexes were predicted with forced pairing at positions 3 and 4 (−f = 3,4) or positions 5 and 6 (–f = 5,6). The predicted structures were usually identical, but when different, the structure with more seed pairing was used. For targets lacking significant seed homology, seed pairing was not forced. To generate duplex structure heat maps, base-paired (Watson-Crick or G-U wobble) miRNA sites were assigned a score of 1, and unpaired sites a score of 0. k-means clustering of the resulting matrix was done with Cluster 3.0 or the stats package in R. Cluster numbers (k) from 3 to 12 were tested, with k = 8 providing the most meaningful set of distinct categories. This assessment was supported by the “elbow” method of optimal k determination. Enrichments of miRNAs in different k groups were evaluated by Fisher’s Exact test, comparing the distribution of each miRNA against the distribution of all interactions.

#### Analysis of miRNA-specific ASC and gene ontology

For miRNA families with >=500 chimeras on 3′ UTRs, ASC values were calculated as

$$ASC={\mathrm{log}}_{2}(\frac{\frac{\sum {\rm{miRf}}\,{\rm{chimeras}}({\rm{r}})}{\sum {\rm{miRf}}\,{\rm{chimeras}}({\rm{t}})}}{{\rm{RPKM}}({\rm{r}})}),$$

where *f* denotes the miRNA family of interest, *r* the mRNA in question and *t* total number.

GO analysis for selected miRNA families was done using the DAVID tool^42^. Genes with ASC values above mean for the given miRNA family (excluding non-targeted mRNAs) were included in the analysis, and all bovine genes were used as background. Functional annotation clustering was done, and names of annotation clusters were manually edited. Annotation clusters with enrichment scores >1 (>1.5 for the miR-27a-3p and let-7a families) were plotted. K-means clustering of genes according to miRNA specific ASC values was done in R in three iterations each using 45 clusters and 100,000 random starts. The 45 clusters were chosen based on the elbow-method and to allow a sufficient number of both single and combined targeting patterns. Clusters with enrichment of specific miRNAs and present in all three iterations were used to build the cooperative binding matrix.

#### Statistical Tests

Statistical analysis was carried out in R or Graphpad Prism. Two-sided KS-Test was employed for CDFs to test both for goodness of fit and a difference in means. Hypergeometric test was employed to evaluate gene overlap. Student’s t-test, linear regression or Fisher’s exact test was used otherwise.

### Availability of data and material

AGO-CLIP and RNA-seq data have been deposited in GEO under accession number GSE90089.

## Electronic supplementary material

Supplementary Information

Supplementary Dataset 1

Supplementary Dataset 2

Supplementary Dataset 3

Supplementary Dataset 4

Supplementary Dataset 5

Supplementary Dataset 6

Supplementary Dataset 7

Supplementary Dataset 8
