# Supplementary material for: Global mapping of miRNA-target interactions in cattle (Bos taurus)
Source: Sci Rep. 2017 Aug 15;7:8190. doi: 10.1038/s41598-017-07880-8 (PMC5557892; doi:10.1038/s41598-017-07880-8)
Supplement: Supplementary file 1 — Supplementary Information [file 41598_2017_7880_MOESM1_ESM.pdf]

Supplementary Material

Global mapping of miRNA-target interactions in cattle (Bos taurus)

Troels K. H. Scheel[1,2,3*], Michael J. Moore[4], Joseph M. Luna[1,4], Eiko Nishiuchi[1], John Fak[4], Robert B. Darnell[4,5], Charles M. Rice[1*]

[1] *Laboratory of Virology and Infectious Disease, Center for the Study of Hepatitis C, The Rockefeller University, New York, NY, USA*

[2] *Copenhagen Hepatitis C Program, Department of Infectious Diseases, Copenhagen University Hospital, Hvidovre, Denmark;*

[3] *Department of Immunology and Microbiology, Faculty of Health and Medical Sciences, University of Copenhagen, Copenhagen, Denmark*

[4] *Laboratory of Molecular Neuro-Oncology, and Howard Hughes Medical Institute, The Rockefeller University, New York, NY, USA*

[5] *New York Genome Center, New York, NY, USA*

* Corresponding authors: Troels Scheel, +45 38622322, tscheel@sund.ku.dk (lead contact); Charles Rice, +1-212-327-7046, ricec@rockefeller.edu

Supplementary Figures

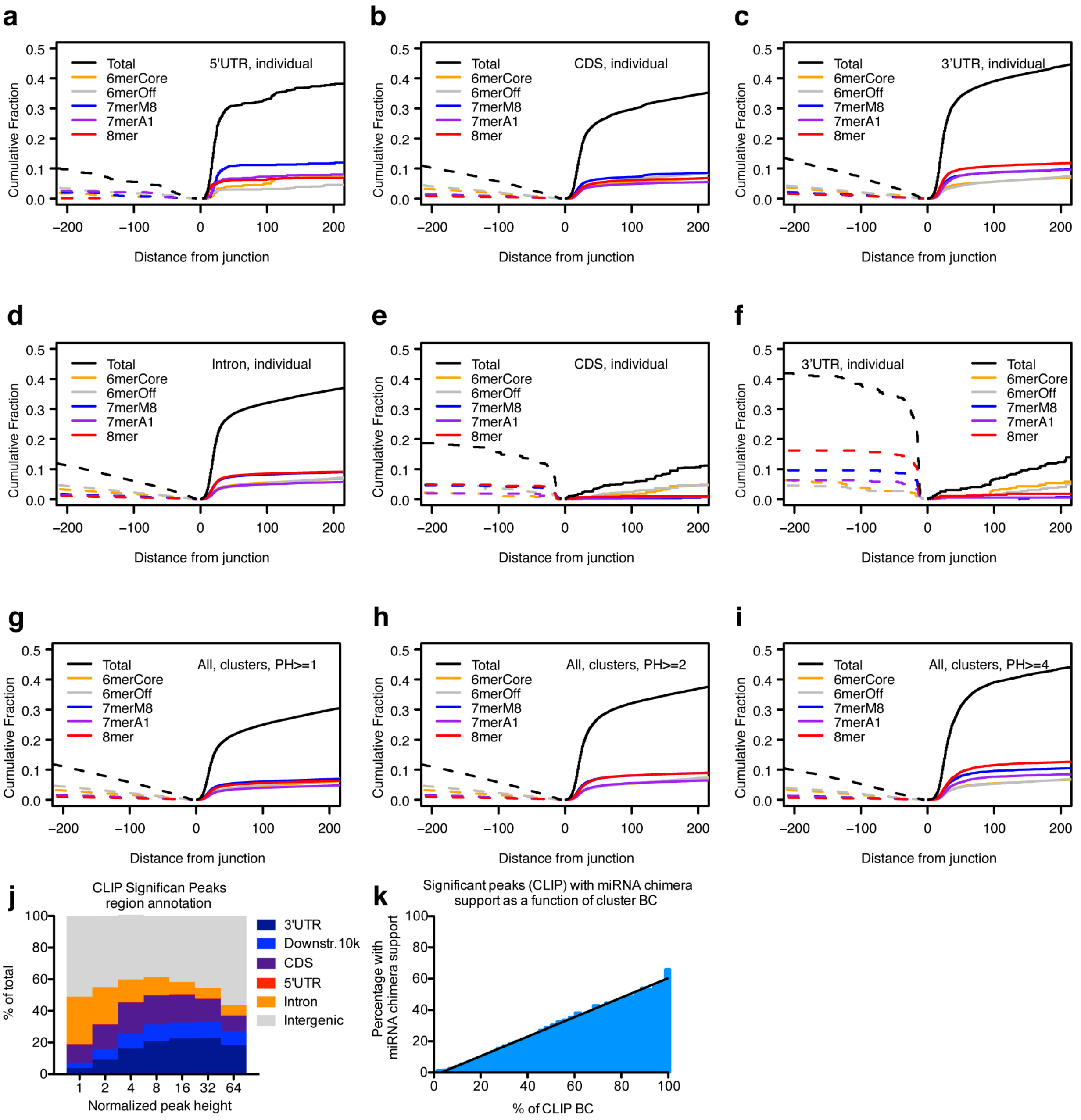

Figure S1. Identification and characterization of miRNA-target chimeras. Related to Figure 1. **(a-d)** Cumulative plots for miRfirst chimeras showing enrichment for canonical miRNA seed sites upstream and downstream in target sequences relative to the chimeric junction site for 5' UTR (a), CDS (b), 3' UTR (c) and intronic regions (d). **(e-f)** Cumulative plots as in (a-d) but for miRlast chimeras for CDS (e) and 3' UTR (f) regions. **(g-i)** Cumulative plots as in (a-d) for miRfirst chimeras broken down by chimera PH. **(j)** Genomic region annotation for significant CLIP peaks as a function of PH. **(k)** Overlap between standard CLIP and miRNA chimera data shown as a function of BC for significant CLIP peaks.

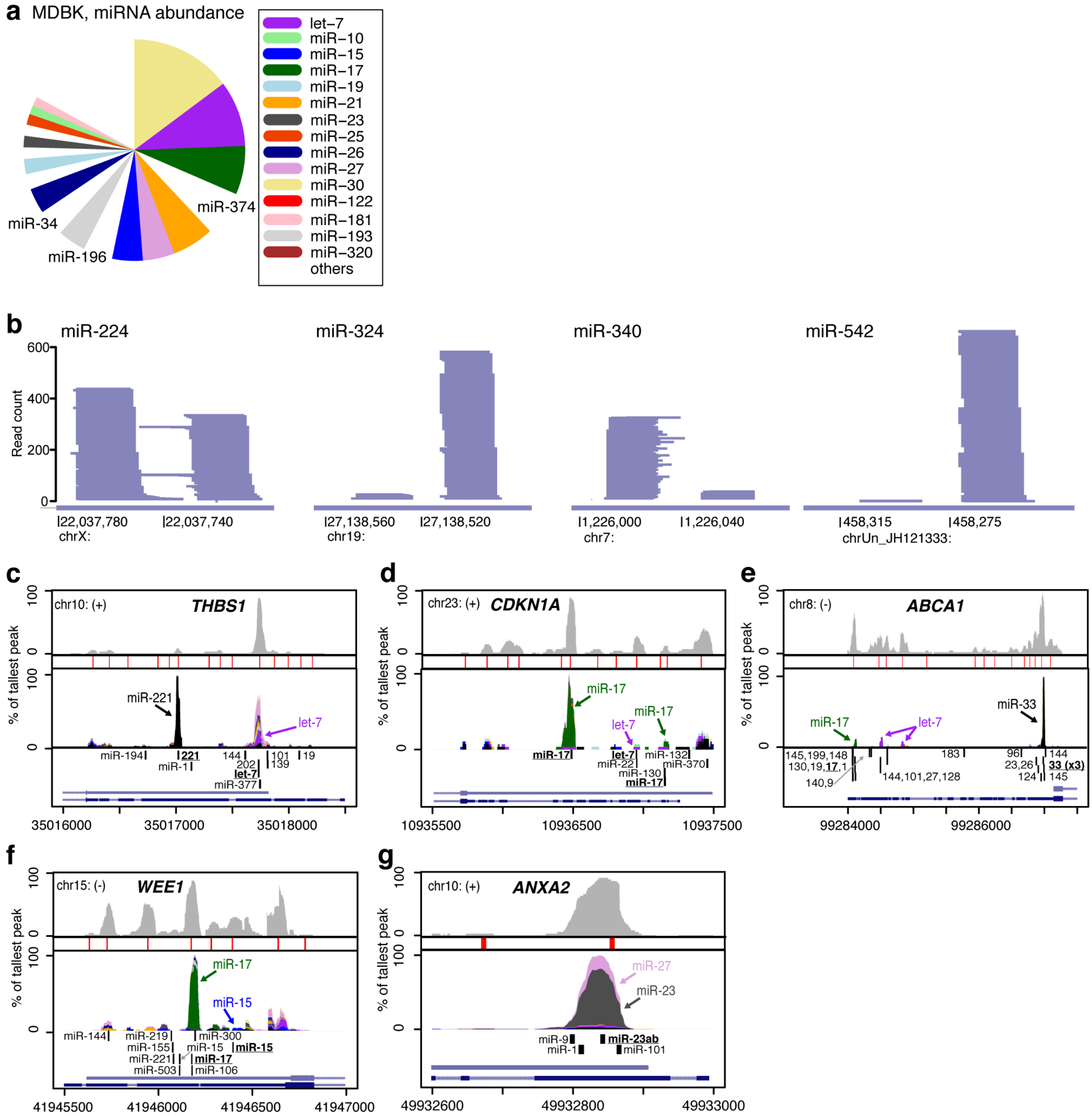

Figure S2. miRNA profile and chimera defined target sites. Related to Figure 2.

(a) Abundance profile of AGO-loaded miRNAs in MDBK cells [1]. **(b)** Mature miRNA expression profiles for four incompletely characterized miRNAs in cattle. Read counts are shown across the miRNA genes of miR-224, -324, -340 and -542. **(c-g)** Specific mRNA 3' UTR profiles of AGO-CLIP data (top, light grey), significant peaks (red bars), miRNA chimera data (middle, colored by miRNA family, see legend in panel [a]), TargetScan predictions (bottom, black bars) and mRNA structure (bottom, light blue: cow; dark blue: human/others).

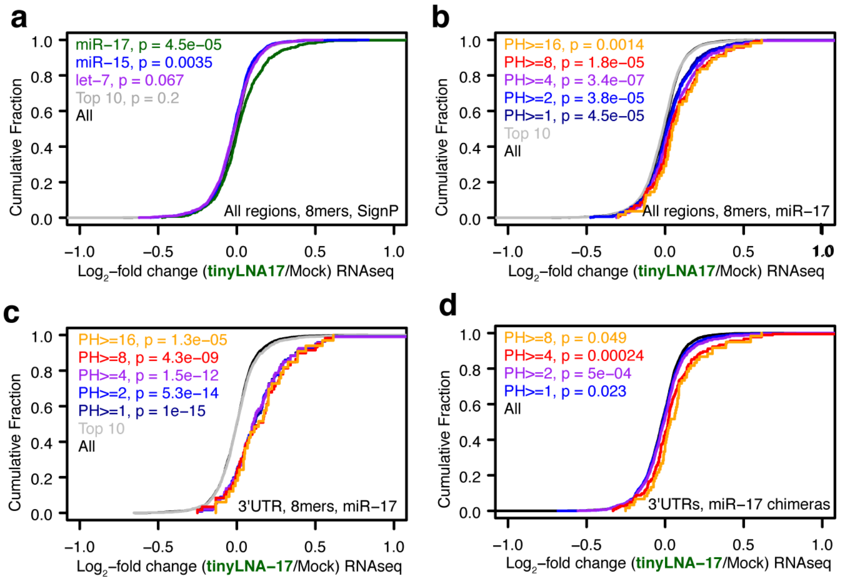

Figure S3. Predictors of miR-17 regulation. Related to Figure 3.

Cumulative density function (CDF) of the $\log_2$-fold change in mRNA abundance between tinyLNA-17 and mock treated MDBK cells is shown using grouping of mRNAs **(a)** by presence of miRNA 8mer seed sites in significant peaks in any genic region, **(b-c)** by presence of miR-17 8mer seed sites in significant AGO-CLIP peaks of increasing peak height in all (b) or 3' UTR (c) regions, or **(d)** by presence of miR-17 chimeras of increasing peak height.

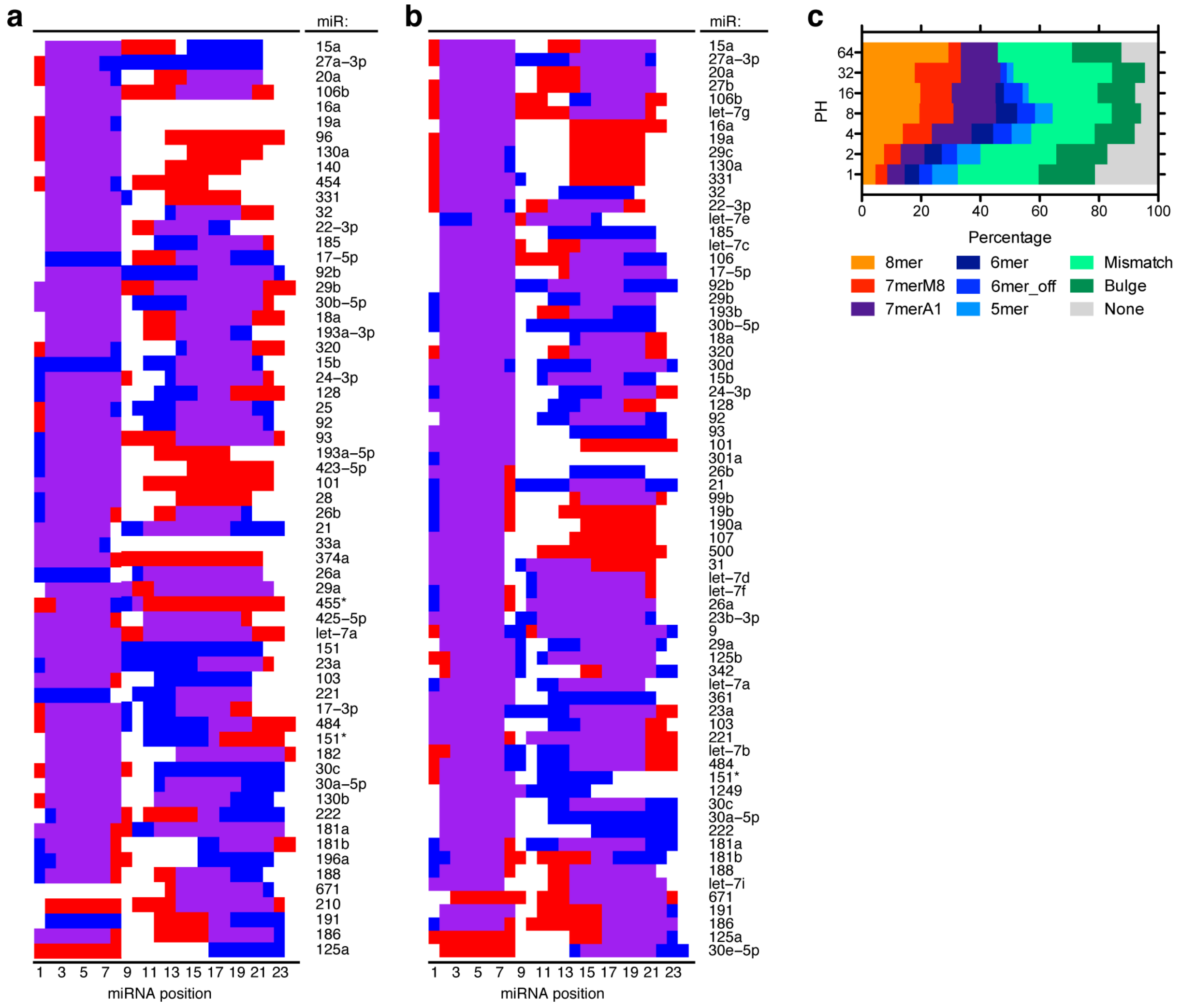

Figure S4. miRNA pairing rules for bovine miRNAs. Related to Figure 4.

(a) Comparative de novo 7mer motif analysis heat map between bovine kidney (blue) and human liver (red) [2] for miRNAs with high enough abundance to be included in both analyses.

(b) Comparative analysis as in (a) but between bovine kidney (blue) and mouse brain (red) [2].

(c) The proportion of chimera-defined target regions with the indicated seed variants is plotted, broken down by chimera PH.

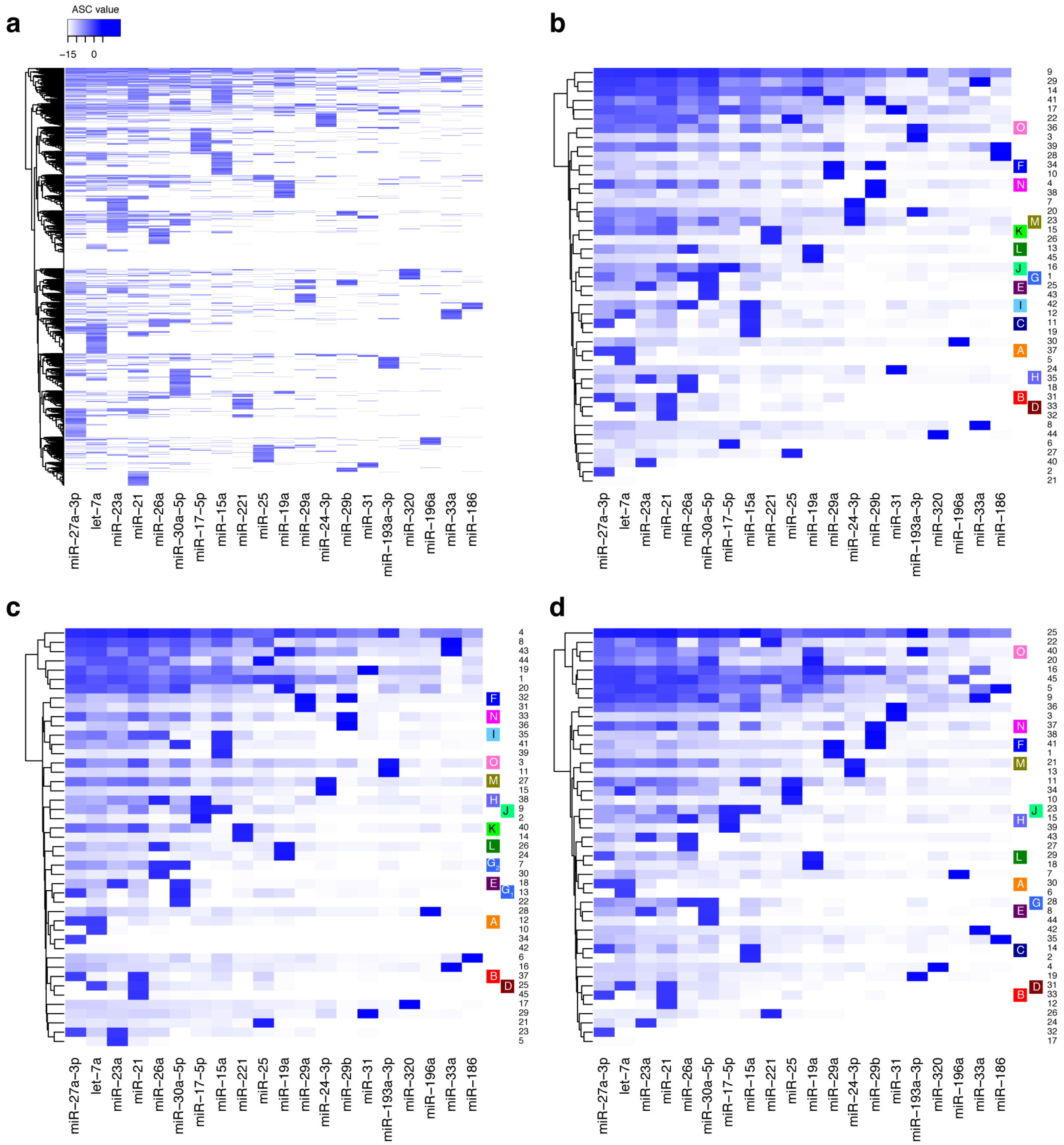

Figure S5. Gene targeting profile for the most abundant miRNAs. Related to Figure 5.

(a) Gene specific ASC values are shown as heatmap for regulation of 5684 bovine mRNAs by the 20 most abundant miRNAs. Genes are organized by hierarchical clustering. High color intensity signifies high ASC value as indicated to the upper left. **(b-d)** Heat maps of mean ASC values in three iterations of k-means clustering of genes by miRNA specific ASC values. Genes were clustered into 45 groups based on their miRNA targeting profile after k-means analysis. Color codes and letter designations correspond to miRNA cooperativity patterns in Fig. 5c.

Supplementary Tables

Table S1. AGO-CLIP alignment statistics.

Note. Alignment statistics are given with indication of CLIP type (CLIP.type), CLIP- and sequence-experimental ID (CLIP.exp; Seq.pool), sequencing index (Index), small RNA treatment (Small.RNA), the number of unique standard CLIP reads aligned to BosTau7 after collapsing for PCR duplicates (Collapsed.reads), the percentage of viral (BVDV) reads in the sample (Virus.reads.perc), the number of miRfirst and miRlast type chimeras (miRfirst; miRlast), the percentage of chimeras compared to total uniquely mapped reads (perc.miRfirst; perc.miRlast) and the estimated "missing" number of total chimeras and miR-17 chimeras (Estim.perc.missing.chim; Estim.perc.missing.miR-17.chim). In the case of BVDV infected samples, these numbers were calculated from virus-specific chimeras. In the case of miR-17 inhibitor treated samples, it was calculated based on the percentage of miR-17 chimeras of total. Provided as an Excel file.

Table S2. Annotation of significant AGO-CLIP peaks, AGO-CLIP read clusters and overlapping miRNA-target chimeras.

Note. 341896 observations of 235 variables. Significant AGO-CLIP peaks (FDR<0.001) are listed and annotated with peak ID (PeakID), chromosome (Chr_SignP), start coordinate (Start_SignP), end coordinate (End_SignP), peak height (PH) and strand (Strand_SignP). In addition are given details for the corresponding AGO-CLIP cluster (all overlapping reads clustered) with chromosome (Chr_Cluster), start coordinate (Start_Cluster), end coordinate (End_Cluster), cluster ID (ClusterName), peak height (PH_Cluster), strand (Strand_Cluster), BC (BC_Cluster), overlap with chimeras in nucleotides (Chim_overlap_Cluster_nt) or presences/absence (Chim_overlap_Cluster), gene ID (GeneID), region annotation (Region) and rmsk annotation (rmsk). Overlap with miRNA-chimera clusters is annotated, including information on chromosome (Chr_Chim), start coordinate (Start_Chim), end coordinate (End_Chim), chimera cluster ID (ChimName), peak height (PH_Chim), strand (Strand_Chim), cognate miRNA (miRNA), start distance between individual chimeras within the chimera cluster

(Start_dist), BC (BC_Chim), gene ID (GeneID_Chim), region (Region_Chim) and miRNA family (Type.miR). Finally, the following annotation is given for the significant peak: gene ID (GeneID_SignP), region (Region_SignP), unique ID for the combined significant peak/cluster/chimera cluster (UniqueID), chromosome length (Chr.len) and DNA sequence (Seq_SignP). Lastly, presence/absence is indicated for 6mer, 7merA1, 7merM8 and 8mer seed sites of the 50 most abundant miRNAs. Provided as compressed csv file.

Table S3. List and annotation of individual miRNA-target chimeras.
Note. 296297 observations of 20 variables. All individual miRNA-target chimeras are listed with gene name (GeneName), gene ID (GeneID), chromosome (Chr), start coordinate (Start), end coordinate (End), read name (ReadName), collapsing factor (PH), strand (Strand), cognate miRNA (miRNA), start coordinate for miRNA within the read (miRstart), start coordinate for target sequence within the read (targetStart), length of target sequence (targetLen), target sequence (targetSeq), chimera sequence (Seq), data set (DataSet), chimera type (Type), region (Region), miRNA family (Type.miR), rmsk overlap in percentage (rmsk_perc) and rmsk (rmsk). Provided as compressed txt file.

Table S4. List of potentially de-orphanized peaks from AGO-CLIP in bovine retina.
Note. Potentially de-orphanized peaks from AGO-CLIP in bovine retina [3] are listed with chromosome (Chromosome), cluster start coordinate (Start), cluster end coordinate (End), cluster name (ClusterName), peak height (Tags), strand (Strand), peak start coordinate (Start.peak), peak end coordinate (End.peak), gene name (Gene.Abbreviation), region (Target.Site.Location), miRNA families supported by >=3 chimeras (Type.miR.3plus) and miRNA families supported by >=1 chimera (Type.miR). Provided as an Excel file.

Table S5. Gene expression changes for tinyLNA-17 treated MDBK cells.
Note. Alignment statistics are given gene-wise for mRNA-seq data, including gene id (Gene_ID), read numbers for mock 1 (Mock1), mock 2 (Mock2), tinyLNA-17 1 (tinyLNA17.1), and tinyLNA-17 2 (tinyLNA17.2), log2 fold change mock/tinyLNA-17 (logFC), log(CPM) (logCPM), p-value (PValue) and FDR (p.adj). Provided as an Excel file.

Table S6. Putative ceRNAs in MDBK cells.

Note. Genomic regions were identified with at least three clusters derived from miRNAs of the same family each consisting of at least five unique chimeras within a genomic region of 3000 bp. RefSeq ID is given for regions annotated as genes in cow. Gene name is in addition given for regions annotated as genes in other species. In addition, region (`Region`), chromosome (`Chr`), start coordinate (`StartPos`), strand (`Strand`), miRNA family (`miRfam`), chimera peak size (`Chimera peak size`), other miRNA peaks of at least 20 chimeras (`Other miRNA peaks >20`) and notes (`Notes`) are given. Provided as Excel file.

Table S7. miRNA-target duplex predictions.

Note. 168790 observations of 38 variables. miRNA-target duplex predictions using RNAhybrid are presented, including the following information on the target sequence; chromosome (`chr`), start coordinate (`start`), end coordinate (`end`), chimera cluster ID (`cluster.ID`), PH (`cluster.size`), strand (`strand`), miRNA (`miRNA`), sequence (`sequence`), sequence motifs as defined by the chimeric miRNA (`8mer, 7merm8, 7merA1, 6mer, 6mer_off, 6merA1, 5mer_1, 5mer_2, 5mer_3`), presence and position of motifs with "-1" indicating absence (`pos.8mer, pos.7merm8, pos.7merA1, pos.6mer, pos.6mer_off, pos.8mer.mm, pos.8mer.indel, pos.7merm8.mm, pos.7merm8.indel, pos.6merA1, pos.5mer_1, pos.7merA1.mm, pos.7merA1.indel, pos.5mer_2, pos.5mer_3, pos.6mer.mm, pos.6mer_off.mm, pos.6mer.indel, pos.6mer_off.indel`), dominant seed type (`which.seed`) and position (`where.seed`).

Table S8. Gene specific ASC values for the 20 most abundant miRNAs.

Note. Gene specific ASC values are given for all miRNAs (any AGO binding) and for the 20 most abundant miRNAs. In addition, RefSeq ID, Ensembl gene ID and transcript ID, gene name and Entrez gene ID are given.

References

1 Scheel, T. K. *et al.* A Broad RNA Virus Survey Reveals Both miRNA Dependence and Functional Sequestration. *Cell host & microbe* **19**, 409-423, doi:10.1016/j.chom.2016.02.007 (2016).

2 Moore, M. J. *et al.* miRNA-target chimeras reveal miRNA 3'-end pairing as a major determinant of Argonaute target specificity. *Nature communications* **6**, 8864, doi:10.1038/ncomms9864 (2015).

3 Sundermeier, T. R. *et al.* Argonaute high-throughput sequencing of RNAs isolated by cross-linking immunoprecipitation reveals a snapshot of miRNA gene regulation in the mammalian retina. *Biochemistry* **53**, 5831-5833, doi:10.1021/bi500966b (2014).
